# Prospective Study of Low-Radiation and Low-Iodine Dose Aortic CT Angiography in Obese and Non-Obese Patients: Image Quality and Impact of Patient Characteristics

**DOI:** 10.3390/diagnostics12030675

**Published:** 2022-03-10

**Authors:** Matthias A. Fink, Sibylle Stoll, Claudius Melzig, Andrea Steuwe, Sasan Partovi, Dittmar Böckler, Hans-Ulrich Kauczor, Fabian Rengier

**Affiliations:** 1 Clinic for Diagnostic and Interventional Radiology, Heidelberg University Hospital, Im Neuenheimer Feld 420, 69120 Heidelberg, Germany; matthias.fink@uni-heidelberg.de (M.A.F.); sibylle.stoll@med.uni-heidelberg.de (S.S.); claudius.melzig@med.uni-heidelberg.de (C.M.); andrea.steuwe@med.uni-duesseldorf.de (A.S.); hans-ulrich.kauczor@med.uni-heidelberg.de (H.-U.K.); 2 Department of Diagnostic and Interventional Radiology, Medical Faculty, University Dusseldorf, 40225 Dusseldorf, Germany; 3 Department of Interventional Radiology, Cleveland Clinic Main Campus, 9500 Euclid Avenue, Cleveland, OH 44195, USA; partovs@ccf.org; 4 Department of Vascular and Endovascular Surgery, Heidelberg University Hospital, Im Neuenheimer Feld 420, 69120 Heidelberg, Germany; dittmar.boeckler@med.uni-heidelberg.de

**Keywords:** aorta, computed tomography angiography, radiation dosage, contrast media, obesity

## Abstract

The purpose of this study was to prospectively analyse image quality and radiation dose of body mass index (BMI)-adapted low-radiation and low-iodine dose CTA of the thoracoabdominal aorta in obese and non-obese patients. This prospective, single-centre study included patients scheduled for aortic CTA between November 2017 and August 2020 without symptoms of high-grade heart failure. A BMI-adapted protocol was used: Group A/Group B, BMI < 30/≥ 30 kg/m^2^, tube potential 80/100 kVp, total iodine dose 14.5/17.4 g. Intraindividual comparison with the institutional clinical routine aortic CTA protocol was performed. The final study cohort comprised 161 patients (mean 71.1 ± 9.4 years, 32 women), thereof 126 patients in Group A (mean BMI 25.4 ± 2.8 kg/m^2^) and 35 patients in Group B (34.0 ± 3.4 kg/m^2^). Mean attenuation over five aortoiliac measurement positions for Group A/B was 354.9 ± 78.2/262.1 ± 73.0 HU. Mean effective dose for Group A/B was 3.05 ± 0.46/6.02 ± 1.14 mSv. Intraindividual comparison in 50 patients demonstrated effective dose savings for Group A/B of 34.4 ± 14.5/25.4 ± 14.1% (both *p* < 0.001), and iodine dose savings for Group A/B of 54/44.8%. Regression analysis showed that female sex and increasing age were independently associated with higher vascular attenuation. In conclusion, BMI-adapted, low-radiation and low-iodine dose CTA of the thoracoabdominal aorta delivers diagnostic image quality in non-obese and obese patients without symptoms of high-grade heart failure, with superior image quality in females and the elderly.

## 1. Introduction

Computed tomography angiography (CTA) is the imaging modality of choice for diagnosis, follow-up, and preoperative planning of most aortic diseases [1,2,3]. Radiation dose and acute kidney injury after CTA remain concerns, especially in patients with cardiovascular disease, who often require lifelong imaging surveillance with repeated CTA acquisitions and frequently have impaired renal function—although recent guidelines suggest that the risk of acute kidney injury after contrast imaging is lower than previously thought [4,5,6]. Furthermore, as CT examinations account for about two-thirds of the total radiation dose of all radiographic examinations in Europe, a reduction in effective dose (ED) of CT examinations will ultimately decrease incidence of cancer attributable to medical imaging [7]. Several strategies have been investigated to reduce radiation and iodine dose of aortic CTA [8,9,10,11,12,13,14,15,16]. The most frequently studied strategies include automated tube current modulation, automated tube potential selection, lowering of tube potential, high-pitch protocols, iterative reconstruction techniques, and combinations thereof [8,10,13,14,15,16]. With technical advances of CT scanner technology, such as tubes with high power, high-pitch protocols or most recent iterative reconstruction algorithms, the potential savings of radiation and iodine dose for aortic CTA have continuously increased. Over the last decade, studies achieved a mean ED of 4.4–9.6 mSv and mean total iodine doses of 10.5–41 g for CTA of the thoracoabdominal aorta and demonstrated diagnostic image quality of low tube potential protocols for non-obese patients [8,9,11,13,14,15]. However, previous studies suggested insufficient image quality of low tube potential protocols for obese patients, specifically excluded patients with a BMI ≥ 30 kg/m^2^, did not report the body weight and body mass index (BMI), or optimised only either radiation or iodine dose [8,9,11,13,14,15]. Our hypothesis was that radiation and iodine dose for aortic CTA could be reduced for both non-obese and obese patients by means of an optimised, BMI-adapted CTA protocol. Thus, the purpose of this study was to prospectively analyse image quality and radiation dose of an optimised, BMI-adapted low-radiation and low-iodine dose CTA protocol for the thoracoabdominal aorta in non-obese and obese patients, and to investigate the impact of patient characteristics on the image quality of the low-dose CTA protocol.

## 2. Materials and Methods

### 2.1. Study Design and Patients

The patients included in the present study are part of a prospective, single-centre, cross-sectional study (www.drks.de, DRKS00013082). Inclusion criteria were an elective, clinically indicated CTA of the thoracoabdominal aorta without the need of ECG synchronisation, age of 18 years or older, and written informed consent. Exclusion criteria were absolute contra-indications to contrast media administration, known pregnancy, emergency, patients with symptoms of high-grade cardiac insufficiency (New York Heart Association [NYHA] class III or IV), patients with acute psychosis or other conditions with impairment of cognitive ability, and patients not able to cooperate. The NYHA class was assessed by means of a simple questionnaire on limitations of physical activity (Supplementary Table S1) [17]. Patients with BMI < 30 kg/m^2^ (normal weight and overweight) were assigned to Group A, patients with BMI ≥ 30 kg/m^2^ (obese) to Group B [18]. In patients with more than one aortic disease, the primary reason for CTA was documented.

### 2.2. Image Acquisition

All low-dose CTA examinations were performed following a BMI-adapted acquisition protocol using a clinical CT scanner (Somatom Definition Flash, Siemens Healthineers) and a double head injector. Scan parameters and the contrast protocol were adjusted for the two BMI groups. Tube potential was set to 80 kVp for Group A, and 100 kVp for Group B. The final scan and contrast protocols were derived in a three-step pilot study, first gradually reducing reference tube current and iodine delivery rate for each group until mean vascular attenuation dropped below 250 Hounsfield units (HU) and noise below 25 HU, then adjusting the flow rate according to the iodine delivery rate, and, finally, reducing the injection time until mean vascular attenuation dropped further. Details of the final protocols are listed in Supplementary Table S2. The double head injector was loaded with contrast medium of 350 mg iodine/mL concentration (Accupaque 350, GE Healthcare) and physiological saline solution, respectively. The contrast medium was diluted with saline resulting in a lower iodine concentration of the contrast bolus. Contrast medium timing was performed using a bolus tracking technique. A circular region of interest was placed within the aortic lumen at the level of the celiac artery. The threshold was set to 120 HU, the delay to 5 s. Image reconstruction parameters were: slice thickness 1.0 mm, I26f kernel, SAFIRE level 3. The institutional clinical routine aortic CTA protocol using automated tube potential selection and tube current modulation served as comparison (Supplementary Table S2).

### 2.3. Image Quality Evaluation

Objective image quality evaluation was performed using a dedicated image evaluation software (Intuition, TeraRecon Inc., Durham, NC, USA). Mean attenuation in HU and standard deviation (SD) were measured using circular regions of interest (ROI). ROIs within the lumen were drawn as large as possible while excluding the aortic wall, thrombus, plaques, calcifications, and dissection membranes. If an aortic dissection was present, the ROI was placed within the true lumen. The following locations were evaluated: ascending and descending aorta at the level of the pulmonary trunk, suprarenal abdominal aorta at the level of the superior mesenteric artery, infrarenal abdominal aorta just above the aortic bifurcation, and right common iliac artery. For each measurement location, an additional circular ROI of 2 cm^2^ was placed on the same axial slice in the centre of the right paraspinal or psoas muscles, respectively. Contrast-to-noise ratio (CNR) and signal-to-noise ratio (SNR) were calculated as follows with noise defined as the SD of the HU attenuation value of the muscle at the respective level [19]:

(1) $$CNR=\frac{mean\;HU\;attenuation_{vessel}-mean\;HU\;attenuation_{muscle}}{Noise}$$

(2) $$SNR=\frac{mean\;HU\;attenuation_{vessel}}{Noise}$$

For assessment of interobserver agreement, all measurements were independently performed by two radiologists with 3 and 2 years of experience in vascular imaging in an individual random order and blinded to any clinical data and any other measurements.

In addition, subjective image quality was independently rated by two board-certified radiologists with 8 and 6 years of experience in vascular imaging in an individual random order and blinded to any clinical data and any other measurements, using a five-point Likert scale: 5 = excellent, 4 = good, 3 = moderate, 2 = fair, 1 = non-diagnostic (Table 1). For examinations with disagreement between the two radiologists, only the lower rating counted rather than the average of both ratings in order to avoid overestimating image quality.

### 2.4. Radiation Dose Evaluation

Volumetric CT dose index (CTDI_vol_) values, dose-length product (DLP), tube potential and scan coverage were extracted from the dose report provided for each CT examination. ED values were calculated by multiplying the DLP with the region-specific conversion coefficient (k) for scans including chest, abdomen and pelvis (k = 0.015 mSv/mGy × cm), as previously described [20,21,22]. Intraindividual comparisons of radiation exposure parameters between the low-dose CTA protocol and the clinical routine CTA protocol were performed in a subpopulation of patients, who had received both CTA protocols with equal scan range, the same CT scanner and the same contrast medium.

### 2.5. Statistical Analysis

All statistical analyses were performed using R version 3.6.2 (R Foundation for Statistical Computing) and SPSS Version 27.0 (SPSS Inc., Chicago, IL, USA). A *p*-value of < 0.05 was considered statistically significant. Interobserver agreement was determined by computing the Shrout and Fleiss intraclass correlation coefficient (ICC) and interpreted as follows: 0–0.20 = slight, 0.21–0.40 = fair, 0.41–0.60 = moderate, 0.61–0.80 = substantial, and 0.81–1.0 = excellent agreement [23]. Two-sided *t*-tests for independent samples or Chi-squared tests were used as appropriate to test for differences between Group A and Group B patients. The two-sided *t*-test for paired samples for normally distributed data and the Wilcoxon signed rank test for paired samples for non-normally distributed date were applied to test for differences of radiation exposure parameters between the low-dose and the clinical routine protocol. The association between attenuation and the patient characteristics age, sex, and diagnosis was analysed by means of multivariate linear regression, including BMI, tube potential, and tube current as potentially confounding covariates. Stepwise inclusion was applied, with a required variable significance of 0.05 to be included into the model, and a cut-off value of 0.1 for exclusion. Significant collinearity between covariates was excluded. Finally, to test for differences between subgroups, analysis of variance and post hoc tests using Bonferroni correction were applied.

## 3. Results

### 3.1. Study Cohort

**Figure 1:**
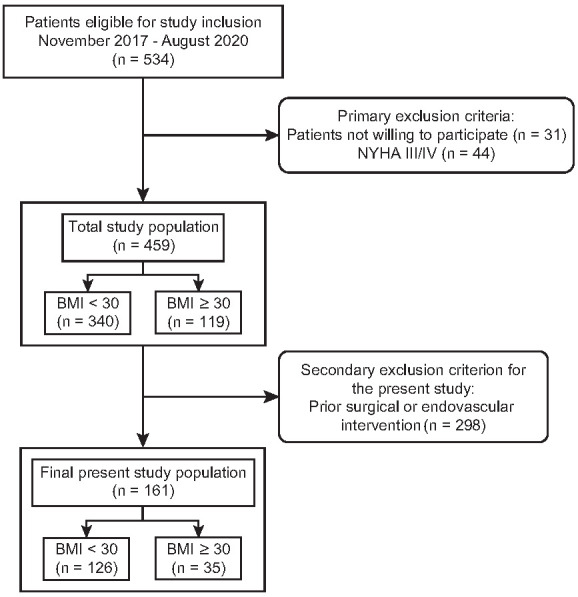
Flowchart of the study.

Between November 2017 and August 2020, until the dismounting of the study CT scanner, 459 patients were enrolled (Figure 1). For the present study, patients with prior aortic surgery or endovascular repair were secondarily excluded, resulting in a final study cohort of 161 patients (mean age, 71.1 ± 9.4 years; 32 female), thereof 126 patients with BMI < 30 kg/m^2^ in Group A and 35 patients with BMI ≥ 30 kg/m^2^ in Group B (Table 2).

The total iodine dose was 14.5 g for Group A and 17.4 g for Group B. Compared to the clinical routine CTA protocol with a total iodine dose of 31.5 g, the total iodine dose saving was 54% for Group A and 44.8% for Group B.

### 3.2. Image Quality Evaluation

**Figure 2:**
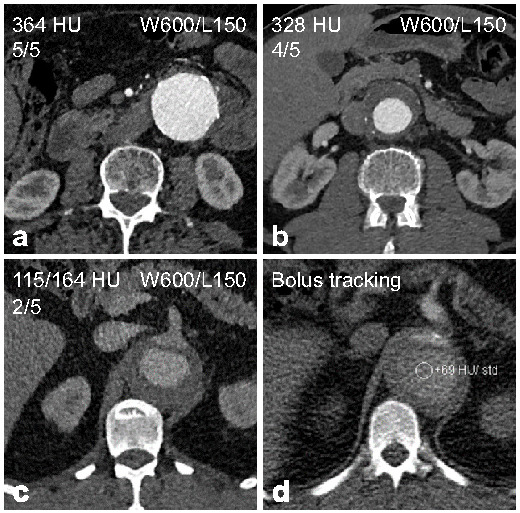
Representative examples for the low-dose CTA with identical window/level (W/L) settings. (**a**) CTA rated as excellent image quality. (**b**) CTA rated as good image quality. (**c**) CTA rated as fair image quality. (**d**) Bolus tracking of the same patient as in **c** showing the monitoring ROI placed in the false lumen of the aortic dissection.

The objective and subjective image quality evaluations are summarised in Table 3 and Table 4. Interobserver agreement was excellent at all locations for HU attenuation (ICC 0.99–1.0, all *p* < 0.001), CNR (ICC, 0.87–0.96; all *p* < 0.001), and SNR measurements (ICC, 0.86–0.94; all *p* < 0.001). Overall, the majority of CTA examinations (89.4%) was rated as having excellent or good image quality (Figure 2). As expected, Group A patients exhibited better objective and subjective image quality compared to Group B patients.

Only one examination (0.6%) was rated as non-diagnostic, a 58-year-old male patient with a BMI of 34.0 kg/m^2^ and aortic dissection. Three examinations (1.9%), all in Group B, were rated as fair image quality, all male patients with a BMI > 30 kg/m^2^, respectively, and all with aortic dissection. Review of these examinations revealed that the bolus tracking region of interest was completely or partially placed within the false lumen of the aortic dissection, leading to a delayed increase in HU attenuation within the region of interest (Figure 2).

### 3.3. Radiation Dose Evaluation

Radiation exposure parameters of the low-dose CTA protocol are summarised in Table 5. Fifty patients had received both the low-dose CTA protocol and the clinical routine CTA protocol with equal scan range, the same CT scanner and the same contrast medium, and thus were included into the intraindividual comparison.

**Figure 3:**
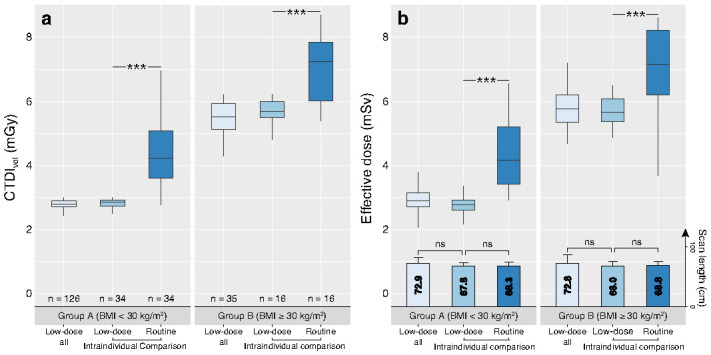
Boxplot diagrams of CTDI_vol_ (**a**) and effective dose (**b**) for the two groups, including bar charts of the scan length (in **b**). Values given for the low-dose CTA in the total study cohort (light blue), the low-dose CTA in the subpopulation of the intraindividual comparison (mid blue) and the clinical routine CTA in the subpopulation of the intraindividual comparison (dark blue). *** *p* < 0.001, ns = not significant. BMI = body mass index, CTDI_vol_ = Volumetric CT dose index.

The time interval between the two CTA examinations varied from 1–61 months (median 12 months). The scan length did not differ between the study CTA protocol (Group A, 67.9 ± 5.6 cm; Group B, 68.0 ± 7.6 cm) and the clinical routine CTA protocol (Group A, 68.4 ± 5.3 cm; 68.1 ± 6.9 cm) (*p* = 0.48 and *p* = 0.89, respectively). The intraindividual comparison demonstrated that the radiation dose was significantly lower for the low-dose CTA protocol compared to the clinical routine CTA protocol, in both Group A and Group B patients (Table 6, Figure 3).

### 3.4. Impact of Patient Characteristics

**Figure 4:**
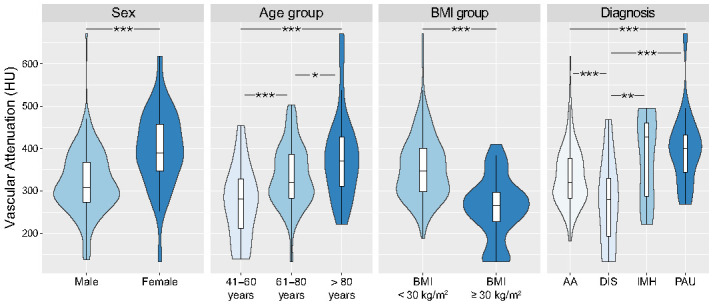
Violin plots and boxplots visualising the subgroup analyses. *** *p* < 0.001, ** *p* = 0.002, * *p* = 0.01. AA = aortic aneurysm, DIS = aortic dissection, IMH = intramural haematoma, PAU = penetrating atherosclerotic ulcer.

Regression analysis showed that sex (*p* < 0.001, standardised beta-coefficient 0.29) and age (*p* = 0.002, standardised beta-coefficient 0.21) were independently associated with vascular attenuation after adjustment for BMI, tube potential, and tube current. The variable diagnosis, including all four aortic diseases, was not significantly associated with vascular attenuation (*p* = 0.09). Subsequent subgroup analyses demonstrated significantly higher vascular attenuation in female patients and older age groups (Figure 4). Furthermore, subgroup analyses revealed significantly lower vascular attenuation in patients with aortic dissection compared to patients with aortic aneurysm, penetrating atherosclerotic ulcer (PAU), or intramural haematoma (IMH).

## 4. Discussion

This prospective study investigated an optimised, BMI-adapted low-radiation and low-iodine dose CTA protocol for the thoracoabdominal aorta including both non-obese patients with BMI < 30 kg/m^2^ and obese patients with BMI ≥ 30 kg/m^2^. The low-dose protocol enabled intraindividual mean ED savings of 34.4% in non-obese and 25.4% in obese patients, and total iodine dose savings of 54% in non-obese and 44.8% in obese patients, compared to the clinical routine protocol with automatic tube potential selection and tube current modulation at the same CT scanner. The mean ED for the total study cohort with a mean BMI of 27.3 ± 4.6 kg/m^2^ was 3.7 ± 1.4 mSv with 72.9 cm scan length. Objective and subjective image quality evaluation demonstrated excellent or good image quality in the vast majority of CTA examinations, with better image quality in Group A (non-obese) compared to Group B patients (obese). Regression analysis revealed that female sex and increasing age were independently associated with higher vascular attenuation after adjustment for BMI, tube potential, and tube current.

Optimising aortic CTA remains a topic of interest, considering that many patients with aortic diseases require lifelong, repeated CTA acquisitions with a large scan range and at the same time are at risk of post-contrast acute kidney injury [4,5,6]. In the present study, we aimed to optimise both radiation and iodine dose, for both non-obese and obese patients. The mean ED of 3.7 mSv in our study is lower compared to the range from 4.4 to 9.6 mSv reported in literature studying similar scanner technology and patient cohorts [8,9,14,19].

Some previous studies suggested contrast injection protocols with individually calculated injection volumes or multilevel protocols, e.g., for every increase of 10 kg body weight [8,14,19,24]. Although such multilevel contrast injection protocols potentially allow for reduced and more reliable contrast dosing, they are prone to mistakes in routine clinical practice based on our experience and do not necessarily result in lower iodine doses compared to fixed contrast injection protocols [9,13,14,25]. Therefore, we studied the reported two-level contrast injection protocol for non-obese and obese patients and matched the adjusted scan parameters for the same two groups, making it easy to implement in clinical practice while also taking BMI into account. The total iodine dose in our study of 14.5 g in non-obese and 17.4 g in obese patients ranges at the lower end of the reported 14–41 g in previous studies for aortic CTA with similar scanner technology and patient cohorts using fixed or multilevel protocols [8,9,14,15]. Several studies with even lower iodine doses of 10.5 g or 12 g iodine excluded obese patients and did not optimise radiation dose [11,13,25]. It is known that simultaneous reduction in radiation and iodine dose can negatively impact image quality in CTA, owing to increased image noise and decreased vascular attenuation [13]. Different injection protocols have been suggested to reduce iodine dose for aortic CTA, and the dilution of contrast medium with saline resulting in a lower iodine concentration of the contrast bolus, as also applied in our study, was previously shown to provide the most consistent vascular attenuation [9]. The mean vascular attenuation of 335 HU, CNR of 10.8, and SNR of 12.8 in our study show good agreement with previous studies measuring image quality parameters on thin-slice reconstructions with vascular attenuation ranging from 306 to 338 HU, CNR from 9.7 to 14.2, and SNR from 7.3 to 18.4 [10,14,15]. Although the majority of CTA examinations in our study exhibited excellent or good image quality, it was lower in obese compared to non-obese patients despite higher radiation and iodine doses. The negative correlation of BMI and weight with image quality is well known [26,27,28]. One non-diagnostic CTA (0.6%) and three CTA examinations with fair image quality (1.9%) occurred in obese male patients with aortic dissection due to the bolus tracking covering the false lumen of the aortic dissection. Subgroup analyses confirmed significantly lower vascular attenuation for aortic dissections compared to other aortic diseases. Consequently, our low-dose protocol should be applied with particular attention in patients with known or suspected aortic dissection, especially regarding the positioning of the monitoring ROI in the true lumen if prior imaging is available and the potential need to start the scan manually. Alternatively, bolus tracking could be performed in the ascending aorta. With that said, the study results suggest that the presented low-dose protocol enables diagnostic image quality with very low radiation and iodine doses in both non-obese and obese patients.

Another interesting finding of our study is that female sex and increasing age were independently associated with higher vascular attenuation. The relationship of sex and age with image quality has been investigated for normal-dose CTA of pulmonary, coronary and lower extremity arteries with similar findings of higher vascular attenuation in female and elderly [29,30,31,32]. Our study revealed that these associations also hold true for low-dose aortic CTA. The reason for this is not clear, but may be due to lower cardiac output and/or differences in body composition, e.g., lower blood volume and lower muscle-to-fat ratio, leading to reduced dilution of injected contrast media [27]. Future studies could investigate personalised low-dose CTA protocols with radiation and iodine doses based on the individual combination of sex, age, and BMI.

This study is subject to some limitations. First, we did not compare vascular attenuation, CNR and SNR between the low-dose and the clinical routine protocol because the purpose of the study was to demonstrate diagnostic image quality of the low-dose CTA protocol for both non-obese and obese patients according to the “as low as reasonably achievable” principle, rather than to proof the equivalence regarding image quality between the low-dose and the clinical routine protocol. Thus, the design of our study including low-dose scanning and contrast protocols was based on the a priori assumption that the objective and subjective image quality parameters would differ between the low-dose and the clinical routine protocol. For this reason, a direct comparison of these parameters between the two protocols was not performed. Second, patients with symptoms of high-grade heart failure were excluded because a reduced cardiac output would lead to lower vascular attenuation [27,33]. Although this exclusion criterion limits general applicability of the low-dose CTA protocol, it is easy to implement into clinical routine with a simple questionnaire estimating NYHA class [17]. Third, the BMI at the time of the reference CTA examination was not recorded. Thus, it could not be verified that the BMI group remained the same between the reference and the low-dose CTA examinations. However, it is very unlikely that there was a systematic trend towards reduced or increased BMI affecting the results. Finally, the present study is a single-centre study with all image acquisitions performed on one scanner with 128-slice technology. The presented protocol may require adjustments for other scanners. Although scanner technologies with more slices are commercially available, they are more expensive and likely less widely in use.

## 5. Conclusions

CTA of the thoracoabdominal aorta can be performed with both very low radiation and very low iodine doses in non-obese and obese patients without symptoms of high-grade heart failure while maintaining diagnostic image quality on 128-slice CT scanner technology by means of an optimised, BMI-adapted CTA protocol. The low-dose protocol enabled significant radiation and iodine dose savings compared to the clinical routine protocol with automated tube potential selection and tube current modulation. Female sex and increasing age were independently associated with higher vascular attenuation. In patients with known or suspected aortic dissection, the low-dose protocol should be applied with particular attention regarding the positioning of the monitoring ROI in the true lumen if prior imaging is available and the potential need to start the scan manually.

## Figures and Tables

**Table 1:** Evaluation criteria for subjective image quality.

Rating	Description
Excellent (5)	Aorta and iliac arteries with excellent enhancement and little noise.
Good (4)	Aorta and iliac arteries with good enhancement and little to moderate noise.
Moderate (3)	Aorta or iliac arteries partially with low to moderate enhancement and/or moderate to high noise.
Fair (2)	Aorta and iliac arteries completely with low enhancement and/or high noise.
Non-diagnostic (1)	Aorta or iliac arteries partially with insufficient enhancement and/or disruptive noise.

Values in parentheses represent a five-point Likert scale.

**Table 2:** Clinical characteristics of the study cohort.

Parameter	All Patients	Group A (BMI < 30 kg/m^2^)	Group B (BMI ≥ 30 kg/m^2^)
Patients	161	126	35
Women	32 (19.9)	28 (22.2)	4 (11.4)
Age (y) ^†^	71.1 ± 9.4	71.7 ± 9.3	69.1 ± 9.4
Weight (kg) ^†^	82.8 ± 16.3	76.9 ± 11.7	104.3 ± 12.3
Height (cm) ^†^	173.9 ± 8.8	173.6 ± 8.9	175.1 ± 8.4
BMI (kg/m^2^) ^†^	27.3 ± 4.6	25.4 ± 2.8	34.0 ± 3.4
History of cardiac disease	72 (44.7)	57 (45.2)	15 (42.9)
Aortic disease			
Aneurysm	115 (71.4)	92 (73.0)	23 (65.7)
Dissection	22 (13.7)	11 (8.7)	11 (31.4)
PAU	17 (10.6)	16 (12.7)	1 (2.9)
IMH	7 (4.3)	7 (5.6)	0 (0)

Unless otherwise specified, data are frequencies; data in parentheses are percentages. ^†^ Data are mean ± standard deviation. BMI = body mass index, PAU = penetrating atherosclerotic ulcer, IMH = intramural hematoma.

**Table 3:** Objective image quality of the low-dose CTA protocol.

Parameter	All Patients	Group A (BMI < 30 kg/m^2^)	Group B (BMI ≥ 30 kg/m^2^)
Vascular attenuation (HU)			
Average	334.7 ± 85.9 (321.3, 348.1)	354.9 ± 78.2 (341.1, 368.7)	262.1 ± 73.0 (237.0, 287.2)
Ascending aorta	314.8 ± 104.7 (298.5, 331.1)	332.5 ± 101.4 (314.6, 350.4)	249.9 ± 91.2 (218.5, 281.3)
Descending thoracic aorta	346.8 ± 98.8 (331.4, 362.2)	368.5 ± 90.6 (352.5, 384.5)	266.9 ± 87.1 (236.9, 296.9)
Abdominal aorta	346.6 ± 88.7 (332.8, 360.4)	367.6 ± 81.5 (353.2, 382.0)	271.1 ± 71.4 (246.5, 295.7)
Aortic bifurcation	340.2 ± 89.2 (326.3, 354.1)	360.7 ± 83.7 (345.9, 375.5)	266.4 ± 67.5 (243.1, 289.7)
Common iliac artery	324.9 ± 85.6 (311.6, 338.2)	344.4 ± 80.8 (330.1, 358.7)	252.8 ± 61.5 (231.6, 274.3)
Contrast-to-noise ratio			
Average	10.8 ± 3.8 (10.2, 11.4)	11.7 ± 3.5 (11.1, 12.3)	7.8 ± 3.2 (6.7, 8.9)
Ascending aorta	11.1 ± 4.8 (10.4, 11.8)	11.9 ± 4.7 (11.1, 12.7)	8.4 ± 4.6 (6.8, 10.0)
Descending thoracic aorta	12.5 ± 4.8 (11.8, 13.2)	13.4 ± 4.4 (12.6, 14.2)	9.1 ± 4.5 (7.5, 10.7)
Abdominal aorta	10.9 ± 3.9 (10.3, 11.5)	11.7 ± 3.8 (11, 12.4)	8.1 ± 3.8 (7.1, 9.1)
Aortic bifurcation	10.1 ± 4.2 (9.4, 10.8)	11.0 ± 4.1 (10.3, 11.7)	7.0 ± 2.6 (6.1, 7.9)
Common iliac artery	9.6 ± 3.9 (9.0, 10.2)	10.4 ± 3.8 (9.7, 11.1)	6.5 ± 2.2 (5.7, 7.3)
Signal-to-noise ratio			
Average	12.8 ± 3.9 (12.2, 13.4)	13.6 ± 3.6 (13.0, 14.2)	9.6 ± 3.3 (8.5, 10.7)
Ascending aorta	13.3 ± 5.0 (12.5, 14.1)	14.1 ± 4.7 (13.3, 14.9)	10.4 ± 4.7 (8.8, 12.1)
Descending thoracic aorta	14.7 ± 4.9 (13.9, 15.5)	15.6 ± 4.5 (14.8, 16.4)	11.1 ± 4.7 (9.5, 12.7)
Abdominal aorta	12.6 ± 4.0 (12.0, 13.2)	13.4 ± 3.9 (12.7, 14.1)	9.8 ± 3.1 (8.7, 10.9)
Aortic bifurcation	11.9 ± 4.0 (11.3, 12.5)	12.8 ± 3.8 (12.1, 13.5)	8.6 ± 2.8 (7.6, 9.6)
Common iliac artery	11.5 ± 4.1 (10.9, 12.1)	12.3 ± 4.0 (11.6, 13.0)	8.2 ± 2.3 (7.4, 9.0)

Data are mean ± standard deviation (95% confidence interval). HU = Hounsfield units.

**Table 4:** Subjective image quality of the low-dose CTA protocol.

Rating	All Patients *n* = 161	Group A (BMI < 30 kg/m^2^) *n* = 126	Group B (BMI ≥ 30 kg/m^2^) *n* = 35
Average *	4.5 ± 0.6	4.6 ± 0.5	4.1 ± 0.9
Excellent	78 (48.4)	69 (54.8)	9 (25.7)
Good	66 (41.0)	49 (38.9)	17 (48.6)
Moderate	13 (8.1)	8 (6.3)	5 (14.3)
Fair	3 (1.9)	0 (0)	3 (8.6)
Non-diagnostic	1 (0.6)	0 (0)	1 (2.9)

Unless otherwise specified, data are frequencies; data in parentheses are percentages. * Data are mean ± standard deviation.

**Table 5:** Radiation dose evaluation of the low-dose CTA protocol.

Parameter	All Patients *n* = 161	Group A (BMI < 30 kg/m^2^) *n* = 126	Group B (BMI ≥ 30 kg/m^2^) *n* = 35
Scan length (cm)	72.9 ± 10.5 (71.3, 74.5)	72.9 ± 9.4 (71.3, 74.6)	72.8 ± 13.7 (68.1, 77.5) ^#^
CTDIvol (mGy)	3.39 ± 1.21 (3.20, 3.58)	2.79 ± 0.28 (2.74, 2.84)	5.55 ± 0.69 (5.31, 5.79) *
DLP (mGy*cm)	246.3 ± 93.0 (231.8, 260.8)	203.2 ± 30.6 (197.8, 208.6)	401.5 ± 74.8 (375.8, 427.2) *
ED (mSv)	3.69 ± 1.40 (3.48, 3.91)	3.05 ± 0.46 (2.97, 3.13)	6.02 ± 1.14 (5.63, 6.41) *

Data are mean ± standard deviation, with 95% confidence intervals in parentheses. * *p* < 0.001 and ^#^ *p* < 0.05 compared to Group A. BMI = body mass index, CTDI_vol_ = Volumetric CT dose index, DLP = dose-length product, ED = effective dose.

**Table 6:** Intraindividual comparison of radiation dose between the low-dose and the clinical routine CTA protocols.

Parameter	All Patients Subset *n* = 50		Group A(BMI < 30 kg/m²) Subset *n* = 34		Group B (BMI ≥ 30 kg/m²) Subset *n* = 16	
**Protocol**	**Low-Dose**	**Routine**	**Low-Dose**	**Routine**	**Low-Dose**	**Routine**
Scan length (cm)	67.9 ± 6.3 (66.1, 69.7)	68.3 ± 5.8 (66.6, 69.9) ^ns^	67.9 ± 5.6 (65.9, 69.8)	68.4 ± 5.3 (66.5, 70.2) ^ns^	68.0 ± 7.6 (63.9, 72.0)	68.1 ± 6.9 (64.4, 71.8) ^ns^
CTDI_vol_ (mGy)	3.72 ± 1.38 (3.33, 4.11)	5.58 ± 2.37 (4.91, 6.26) *	2.80 ± 0.19 (2.74, 2.87)	4.44 ± 1.0 (4.09, 4.79) *	5.67 ± 0.43 (5.44, 5.90)	8.02 ± 2.61 (6.63, 9.41) *
DLP (mGy*cm)	252.6 ± 97.3 (224.9, 280.2)	382.0 ± 166.3 (334.7, 429.3) *	190.3 ± 21.6 (182.8, 197.9)	304.4 ± 76.4 (277.7, 331.0) *	384.8 ± 49.6 (358.3, 411.2)	546.9 ± 186.8 (447.4, 646.4) *
ED (mSv)	3.78 ± 1.46 (3.37, 4.20)	5.72 ± 2.5 (5.01, 6.43) *	2.85 ± 0.32 (2.74, 2.97)	4.57 ± 1.15 (4.17, 4.97) *	5.76 ± 0.79 (5.33, 6.18)	8.19 ± 2.83 (6.68, 9.69) *
CTDI_vol_ saving (%)	31.2 ± 14.5 (27.1, 35.3)		34.0 ± 13.7 (29.3, 38.8)		25.1 ± 14.8 (17.2, 33.0)	
ED saving (%)	31.5 ± 14.9 (27.3, 35.8)		34.4 ± 14.5 (29.3, 39.5)		25.4 ± 14.1 (17.9, 32.9)	

Data are mean ± standard deviation, with 95% confidence intervals in parentheses. * *p* < 0.001 and ns (not significant) compared to the low-dose CTA protocol. BMI = body mass index, CTDI_vol_ = Volume CT dose index, DLP = dose-length product. ED = effective dose.

## Data Availability

All relevant data are within the paper and its Supplementary Information. Please contact the corresponding author for further requests.

## Supplementary Materials

The following are available online at https://www.mdpi.com/article/10.3390/diagnostics12030675/s1, Table S1: Questionnaire for estimation of NYHA class. Table S2: Summary of CT protocol parameters.

## Abbreviations

The following abbreviations are used in this manuscript:

BMI	Body mass index
CNR	Contrast-to-noise ratio
CTDI_vol_	Volumetric CT dose index
DLP	Dose-length product
ED	Effective dose
ICC	Intraclass correlation coefficient
IMH	Intramural haematoma
NYHA	New York Heart Association
PAU	Penetrating atherosclerotic ulcer
SNR	Signal-to-noise ratio
