# Supplementary material for: Prospective Study of Low-Radiation and Low-Iodine Dose Aortic CT Angiography in Obese and Non-Obese Patients: Image Quality and Impact of Patient Characteristics
Source: Diagnostics (Basel). 2022 Mar 10;12(3):675. doi: 10.3390/diagnostics12030675 (PMC8947155; doi:10.3390/diagnostics12030675)
Supplement: Supplementary file 1 [file diagnostics-12-00675-s001.zip › diagnostics-1608139-supplementary.pdf]

# Supplementary Information

## Supplementary Table S1

**Questionnaire for estimation of NYHA class.**

| Question | Interpretation |
|---|---|
| Have you been diagnosed with a heart disease?<br>Yes / No | |
| If yes, please choose one of the following which best describes your normal level of activities: | |
| I can undertake all ordinary physical activities without undue fatigue, shortness of breath or heart palpitations, e.g., walk briskly upstairs more than two floors, jog, walk uphill or long distances, swim, ski, shovel snow, spade soil. | NYHA I |
| I feel undue fatigue, shortness of breath or heart palpitations with ordinary physical activities, , e.g., walk briskly upstairs more than two floors, jog, walk uphill or long distances, swim, ski, shovel snow, spade soil. | NYHA II |
| I feel undue fatigue, shortness of breath, heart palpitations or chest pain with less than ordinary physical activities, e.g. climb one flight of stairs at normal pace without stopping, fast walking on level ground. | NYHA III |
| I feel undue fatigue, shortness of breath or heart palpitations at rest, increasing with any physical activity. | NYHA IV |

## Supplementary Table S2

**Summary of CT protocol parameters.**

| Parameter | Group-A protocol | Group-B protocol | Routine protocol |
|---|---|---|---|
| **CT scan** | | | |
| Collimation | 128 x 0.6 | 128 x 0.6 | 128 x 0.6 |
| Tube voltage ($kV_p$) | 80 | 100 | Automatic tube voltage selection (80-120 $kV_p$) |
| Reference tube current (mAs) | 210 | 123 | 239 (at 80 $kV_p$) |
| Automatic tube current modulation | Yes | Yes | Yes |
| Pitch | 1.2 | 1.2 | 1.2 |
| **Image reconstruction** | | | |
| Iterative reconstruction | SAFIRE, level 3/5 | SAFIRE, level 3/5 | None |
| Kernel | I26f | I26f | B20f |
| Slice thickness (mm) | 1.0 | 1.0 | 1.0 |
| Slice increment (mm) | 0.7 | 0.7 | 0.7 |
| **Contrast medium** | | | |
| Contrast medium iodine concentration (mg*I/ml) | 350 | 350 | 350 |
| Bolus injection volume (ml) | 54 | 60 | 90 |
| Thereof saline (ml, %) | 12.4 (23%) | 10.2 (17%) | 0 (0%) |
| Bolus iodine concentration (mg*I/ml) | 269 | 292 | 350 |
| Injection rate (ml/s) | 3.7 | 4.1 | 4.0 |
| Injection time (s) | 14.5 | 14.5 | 22.5 |
| Iodine delivery rate (g*I/s) | 1.0 | 1.2 | 1.4 |
| Total iodine dose (g*I) | 14.5 | 17.4 | 31.5 |
| Total iodine dose saving | 54.0% | 44.8% | - |
| Saline chaser (ml) | 30 | 30 | 40 |
